# Tumor suppressor DCAF15 inhibits epithelial-mesenchymal transition by targeting ZEB1 for proteasomal degradation in hepatocellular carcinoma

**DOI:** 10.18632/aging.202823

**Published:** 2021-04-04

**Authors:** Xiao Dong, Yang Han, Encheng Zhang, Yuqi Wang, Pingzhao Zhang, Chenji Wang, Lin Zhong, Qi Li

**Affiliations:** 1 Department of Oncology, Shanghai General Hospital, Shanghai Jiao Tong University School of Medicine, Shanghai 200080, China; 2 Institute of Translational Medicine, Shanghai General Hospital, Shanghai Jiao Tong University School of Medicine, Shanghai 201620, China; 3 Department of Urology, Shanghai General Hospital, School of Medicine, Shanghai Jiao Tong University School of Medicine, Shanghai 200080, China; 4 State Key Laboratory of Genetic Engineering, Collaborative Innovation Center for Genetics and Development, School of Life Sciences, Fudan University, Shanghai 200438, China; 5 Department of Hepatobiliary and General Surgery, Shanghai General Hospital, Shanghai Jiao Tong University School of Medicine, Shanghai 200080, China

**Keywords:** DCAF15, ZEB1, ubiquitination, epithelial-mesenchymal transition, hepatocellular carcinoma

## Abstract

Epithelial-mesenchymal transition (EMT) is an evolutionarily conserved developmental program that has been implicated in tumorigenesis and confers metastatic properties upon cancer cells. ZEB1 is a master transcription factor that activates the EMT process in various cancers. ZEB1 is reportedly degraded through the ubiquitin proteasome pathway, but the underlying molecular mechanism of this process remains largely unknown in hepatocellular carcinoma (HCC). Here, we identified ZEB1 as a substrate of the CRL4-DCAF15 (DDB1 and CUL4 associated factor 15) E3 ubiquitin ligase complex. DCAF15 acts as an adaptor that specifically recognizes the N-terminal zinc finger domain of ZEB1, then triggers its degradation via the ubiquitin-proteasome pathway. DCAF15 knockdown led to upregulation of ZEB1 and activation of EMT, whereas overexpression of DCAF15 suppressed ZEB1 and inhibited EMT. DCAF15 knockdown also promoted HCC cell proliferation and invasion in a ZEB1-dependent manner. In HCC patients, low DCAF15 expression was predictive of an unfavorable prognosis. These findings reveal the distinct molecular mechanism by which DCAF15 suppresses HCC malignancy and provides insight into the relationship between the CUL4-DCAF15 E3 ubiquitin ligase complex and ZEB1 in HCC.

## INTRODUCTION

Hepatocellular carcinoma (HCC) is a highly aggressive malignant neoplasm and is the fifth leading cause of cancer-related death worldwide [1]. Hepatitis, liver cirrhosis and tumor occurrence are common indications during the development of HCC [2]. Therapeutic strategies used to treat HCC entail both interdisciplinary and multidisciplinary approaches, including surgical resection, liver transplantation, radiofrequency ablation, chemotherapy, targeted medicine and immunological therapy [3]. Nevertheless, the prognosis for HCC remains very poor, with a five-year survival rate that is below 12% [4]. The molecular mechanisms underlying HCC development and progression remain unclear. Consequently, there is an urgent need to identify specific biomarkers of HCC that will enable diagnosis early enough to improve prognosis.

Ubiquitin is covalently attached to substrate proteins through a cascade involving three enzymes: ubiquitin-activating enzyme (E1), ubiquitin-conjugating enzyme (E2) and ubiquitin-ligating enzyme (E3) [5]. The human genome encodes nearly 600 E3 ligases, including seven Cullin proteins (CUL1, CUL2, CUL3, CUL4A, CUL4B, CUL5, CUL7), which mediate nucleation events leading to the formation of a multi-subunit ubiquitin ligase [6]. DDB1 and CUL4 associated factor 15 (DCAF15) is a core component of the Cullin-RING E3 ligases-DCAF15 E3 ubiquitin ligase complex. This complex is composed of CUL4, DCAF15, DDB1 and DDA1 [7]. The Cullin 4 RING ligase (CRL4) contains two homogenous scaffolds, CUL4A and CUL4B, which bind DDB1 and the catalytic subunit RING-finger protein through its N-terminus and C-terminus, respectively [8]. The majority of CUL4A resides in the cytoplasm and regulates substrate ubiquitination; however, low levels of CUL4A are found in the nucleus, where it targets the nuclear proteins HOXA9 and p27 [9, 10]. DDB1 acts as a bridging factor between the Cullin scaffold and its substrate recognition subunit [11]. Earlier reports demonstrated that DDA1 directly binds to amino acids 390-460 of DCAF15 [12] and that DCAF proteins prompt E3 ubiquitin ligase complexes to ubiquitinate and target for degrading key substrates, including ZFP9, IKZF1, IKZF3 and RBM39 [7, 13, 14]. In addition, three aryl sulfonamides, E7820, indisulam and tasisulam, have been shown to induce degradation of RBM39 through recruitment of the CUL4-DDB1-DDA1-DCAF15 E3 ligase complex [15].

ZEB1 is a transcriptional regulator of epithelial-mesenchymal transition (EMT) [16]. To better understand the mechanism by which ZEB1 is degraded through the ubiquitin proteasome pathway, in the present study we surveyed a protein-protein interaction database to determine whether ZEB1 interacts with DCAF family proteins and assess its impact on the proliferation and invasiveness of HCC cells and the EMT process. Our findings provide a model for understanding the role of a DCAF15-ZEB1-EMT axis during the development of HCC.

## RESULTS

### DCAF15 forms a complex with ZEB1 in cells

**Figure 1:**
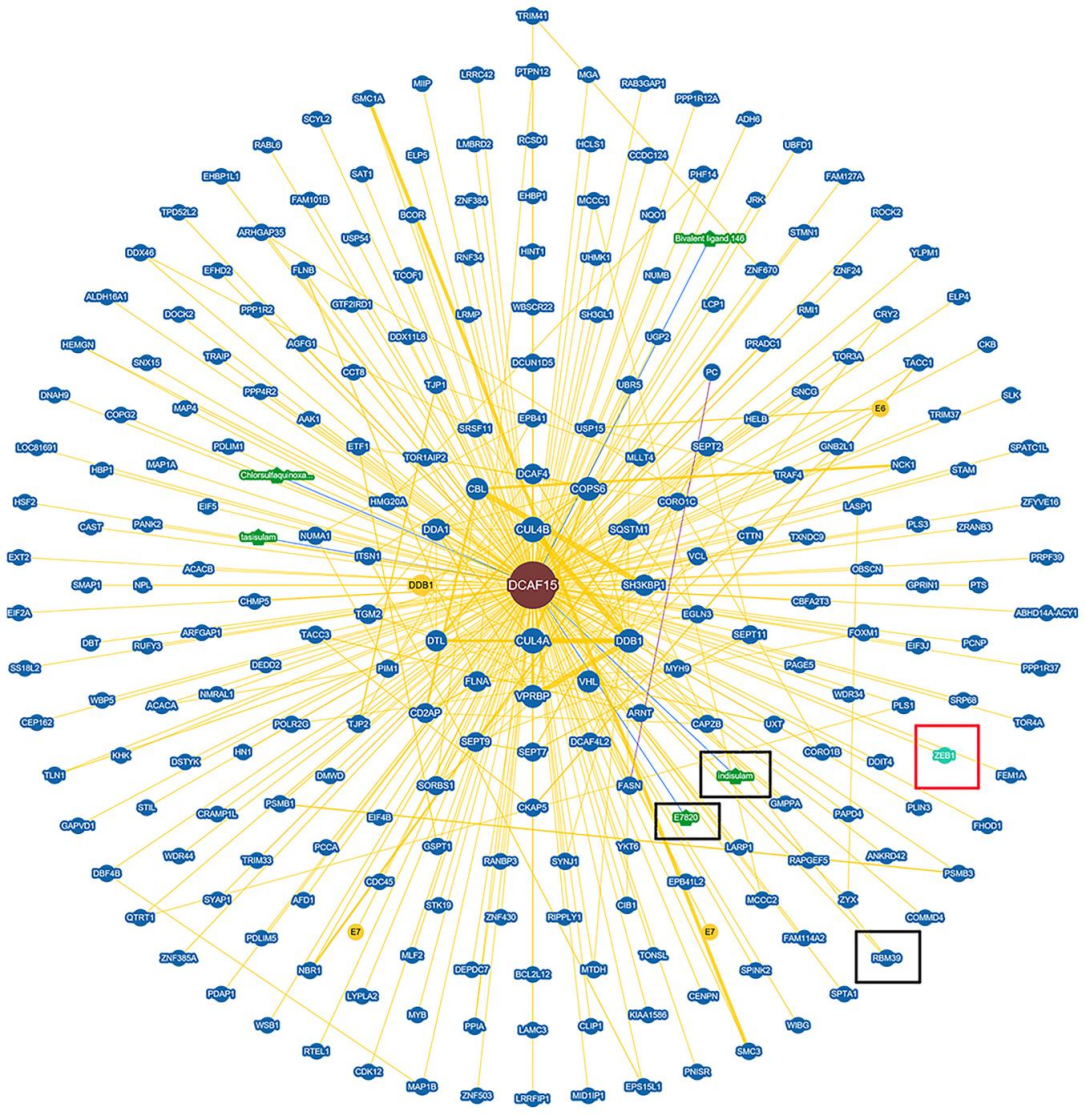
**Protein network diagram shows potential interaction of DCAF15.** Black frame indicates the interactors of DCAF15 reported. Red frame indicates ZEB1 is a potential interactor of DCAF15.

**Figure 2:**
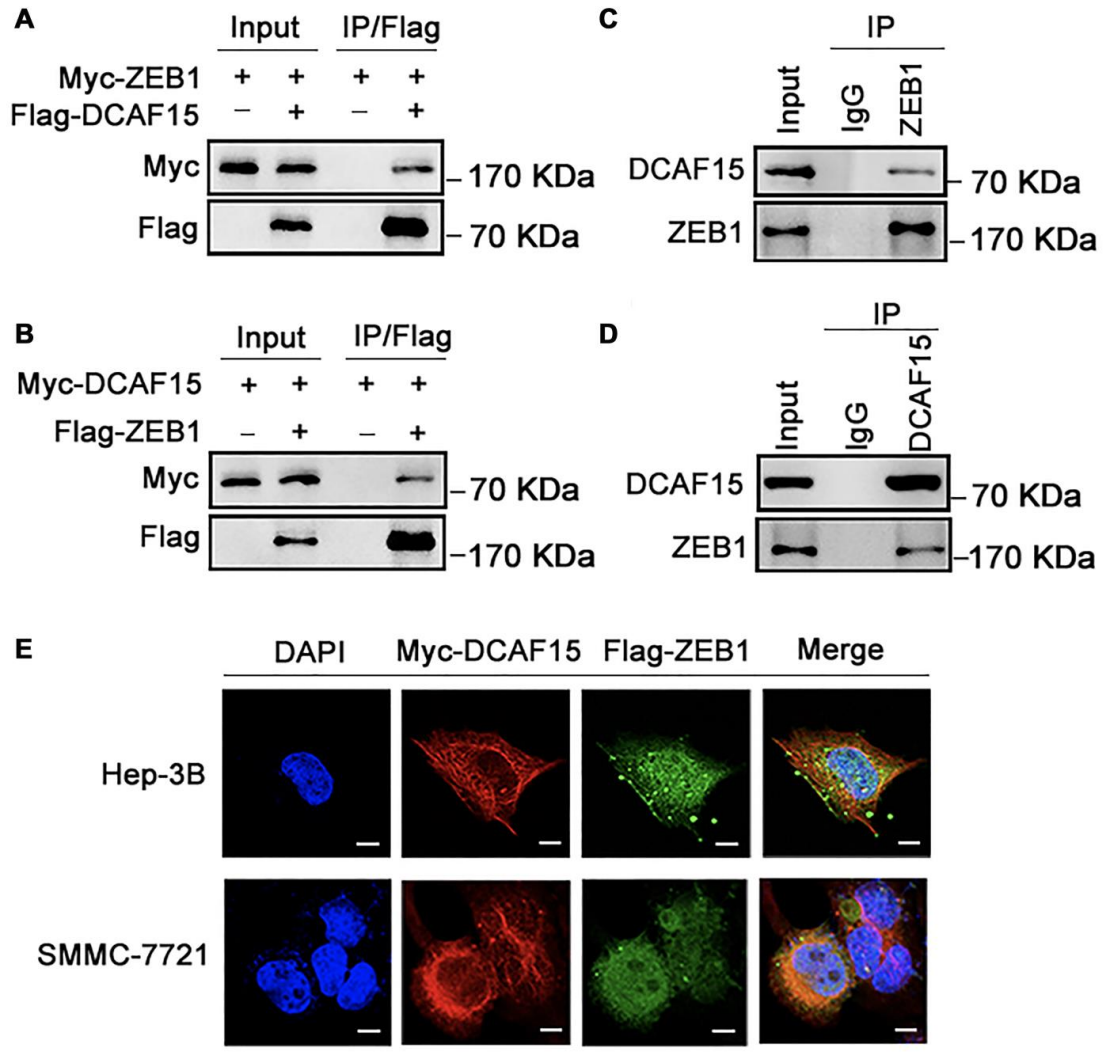
**DCAF15 binds with ZEB1 protein.** (**A**–**B**) Ectopically expressed DCAF15 and ZEB1 can bind with each other. (**A**) Flag-DCAF15 and Myc-ZEB1 were co-expressed in HEK293T cells, and immunoprecipitated with FLAG-M2 agarose beads. (**B**) Similar Co-IP assay was performed between Flag-ZEB1 and Myc-DCAF15. (**C**–**D**) Endogenous interaction between DCAF15 and ZEB1. (**C**) SMMC-7721 cells were treated with 10μM MG132 for 6h. Cell lysates were precipitated using anti-ZEB1 antibody or with IgG (mock IP) and coprecipitated DCAF15 was detected by western blotting. (**D**) SMMC-7721 cells were treated with 10 μM MG132 for 6h. Cell lysates were precipitated using anti-DCAF15 antibody or with IgG (mock IP) and coprecipitated ZEB1 was detected by western blotting. (**E**) Flag-DCAF15 and Myc-ZEB1 were co-expressed in Hep3B and SMMC-7721 cells respectively and visualized by fluorescence microscopy. Scale bars, 30 μm.

To identify the molecular partners interacting with ZEB1, we surveyed a large-scale protein-protein interaction database (https://thebiogrid.org/124705/summary/homo-sapiens/dcaf15.html) and noted that ZEB1 (red frame) is a potential interactor of DCAF15(Figure 1). We then co-expressed Flag-DCAF15 and Myc-ZEB1 in 293T cells, after which the cells were harvested for co-immunoprecipitation (Co-IP) assays. The results indicated that Myc-ZEB1 was immunoprecipitated by Flag-DCAF15 (Figure 2A). Similarly, reciprocal Co-IP assays performed after co-transfecting cells with Flag-ZEB1 and Myc-DCAF15 showed that Myc-DCAF15 was immunoprecipitated by Flag-ZEB1 (Figure 2B), indicating exogenous interaction between these two proteins. We also observed that endogenous DCAF15 was efficiently immunoprecipitated by ZEB1 (Figure 2C) and that endogenous ZEB1 was efficiently immunoprecipitated by DCAF15 (Figure 2D), suggesting there is also endogenous interaction between ZEB1 and DCAF15.

To further investigate the intracellular interaction between ZEB1 and DCAF15, we examined their subcellular localization in two HCC cell lines (Hep-3B and SMMC-7721). After transfecting the cells with Flag-ZEB1 and Myc-DCAF15, they were immunofluorescence-stained with anti-Flag or anti-Myc antibodies. Upon visualization under a microscope, we observed that the exogenous ZEB1 and DCAF15 colocalized in the cytoplasm (Figure 2E). Taken together, these results strongly suggest that DCAF15 forms a complex with ZEB1 within the cytoplasm of HCC cells.

### DCAF15 ubiquitinates and degrades ZEB1 protein

**Figure 3:**
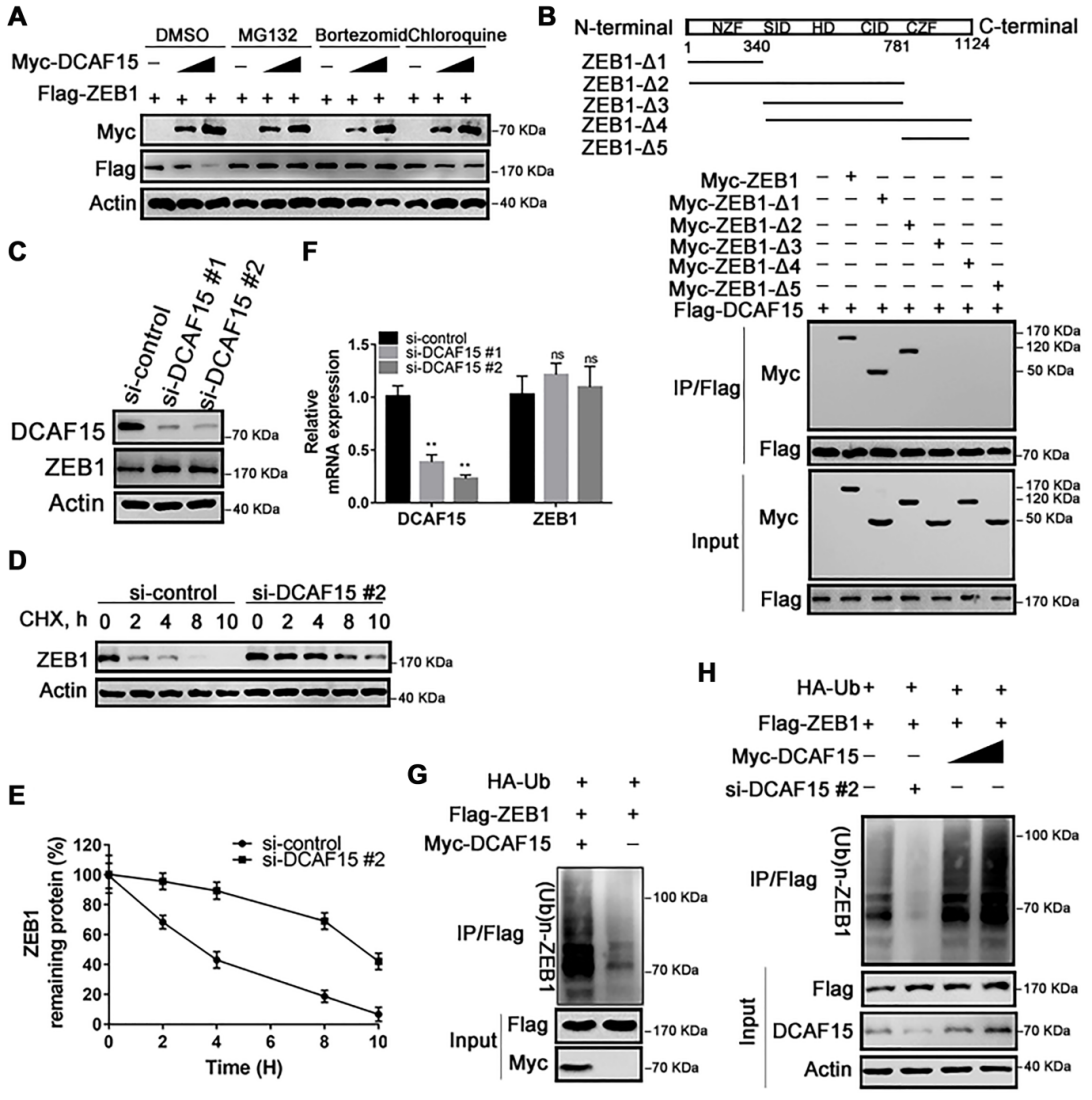
**DCAF15 ubiquitinates and degrades ZEB1 protein.** (**A**) DCAF15 regulates protein level of ZEB1 through the proteasome pathway. HEK293T cells were co-transfected with Flag-ZEB1 and increasing amounts of Myc-DCAF15. After 24h, cells were treated with DMSO, MG132, bortezomib or chloroquine respectively and detected by western blot. (**B**) HEK293T cells were transfected with Flag-DCAF15 and Myc-full-length or truncated ZEB1. After 24 h, cell lysates were immunoprecipitated using FLAG-M2 agarose beads. Immune complexes were analyzed by immunoblotting with the indicated antibodies (lower panel). Schematic diagram illustrating ZEB1 deletion mutants (upper panel). (**C**) SMMC-7721 cells were transfected with the control or two independent DCAF15 siRNAs, respectively. Cell lysates were analyzed via immunoblotting with the indicated antibodies. (**D**) SMMC-7721 cells were transfected with the control or DCAF15 siRNAs. After 24 h, cells were treated with cycloheximide (CHX) for indicated time-periods. Cell lysates were analyzed via immunoblotting with the indicated antibodies. (**E**) The relative intensities of ZEB1 protein were first normalized to the intensities of actin and then normalized to the value of the 0 h point. (**F**) qPCR validation of relative DCAF15 and ZEB1 mRNA levels in DCAF15 control and knockdown cells. GAPDH was used for normalization. (**G**) HEK293T cells were co-transfected with Flag-ZEB1, Myc-DCAF15 and HA-Ub. After 24 h, cells were treated with 10μM MG132 for 6 h. Cell lysates were immunoprecipitated using FLAG-M2 agarose beads and analyzed by immunoblotting with the indicated antibodies. (**H**) DCAF15 promotes ZEB1 ubiquitination. HEK293T cells were co-transfected with Flag-ZEB1, HA-Ub, si-DCAF15 #2 and increasing amounts of Myc-DCAF15. After 24h, cells were treated with MG132. The ZEB1 protein was immunoprecipitated using FLAG-M2 agarose beads. The ubiquitinated ZEB1 was analyzed by Western blotting.

We next investigated whether DCAF15 promotes the ubiquitination and subsequent degradation of ZEB1 protein. Consistent with that idea, levels of Flag-ZEB1 were dose-dependently decreased by expression of Myc-DCAF15, and that effect was absent when cells were treated with a proteasomal inhibitor (MG132, Bortezomib or chloroquine). That all three inhibitors tested rescued ZEB1 from DCAF15-mediated degradation indicates that DCAF15 downregulates ZEB1 levels via the proteasomal degradation pathway (Figure 3A). To identify which ZEB1 domain interacts with DCAF15, we generated five ZEB1 deletion mutants: ZEB1-Δ1 (amino acids 1-340), ZEB1-Δ2 (amino acids 1-781), ZEB1-Δ3 (amino acids 341-781), ZEB1-Δ4 (amino acids 341-1,124) and ZEB1-Δ5 (amino acids 782-1124) [17]. Subsequent Co-IP experiments revealed that the N-terminal zinc finger domain mediated interaction between ZEB1 and DCAF15, but not the Smad-binding domain, homeodomain, CtBP interaction domain, or C-terminal zinc finger domain (Figure 3B). In addition, knocking down endogenous DCAF15 using two targeted siRNAs increased endogenous ZEB1 levels (Figure 3C) and markedly prolonged the half-life of endogenous ZEB1 in SMMC-7721 cells (Figure 3D and 3E).

To address the possibility that the elevated ZEB1 expression was mediated at the transcriptional level, we measured levels of DCAF15 and ZEB1 mRNA in SMMC-7721 cells after transiently treating them with DCAF15 siRNAs. We found that levels of DCAF15 mRNA were significantly decreased, while levels of ZEB1 mRNA were little changed from control, indicating that the degradative effect of DCAF15 on ZEB1 is not mediated at the transcriptional level (Figure 3F). Instead, co-expression of HA-ubiquitin, Flag-ZEB1 and Myc-DCAF15 in HEK293T cells revealed that ZEB1 was robustly and dose-dependently poly-ubiquitinated (Figure 3G and 3H). These results suggest that DCAF15 triggers the ubiquitin-dependent proteasomal degradation pathway through recognition of the N-terminal zinc finger domain of ZEB1 protein.

**Figure 4:**
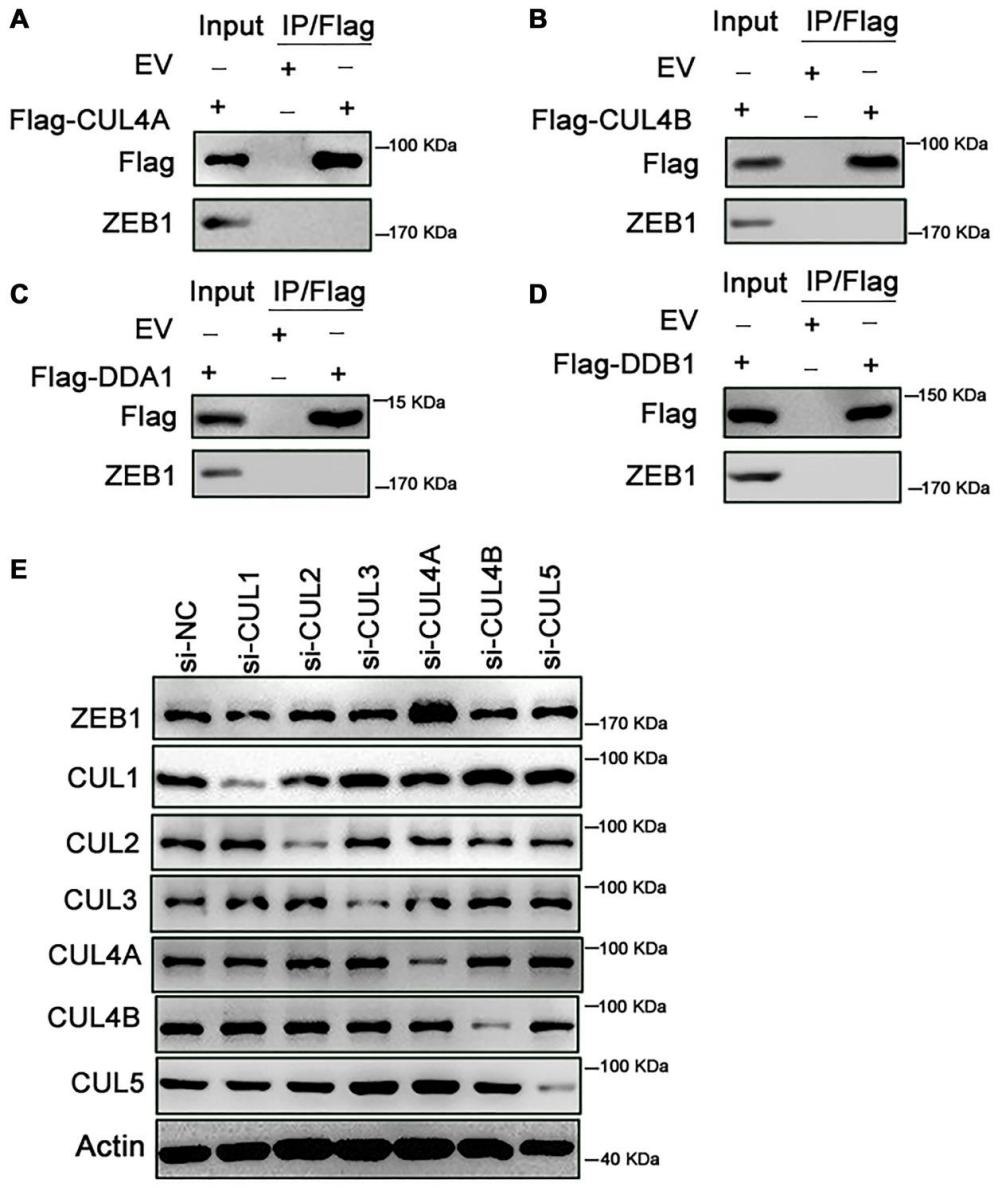
**Knockdown of CUL4A promotes the stability of ZEB1**. (**A**–**D**) HEK293T cells were transfected with Flag-CUL4A, Flag-CUL4B, Flag-DDA1 and Flag-DDB1 respectively. After 24h, cells were treated with 10 μM MG132 for 6h. Cell lysates were immunoprecipitated using FLAG-M2 agarose beads and analyzed by immunoblotting with the indicated antibodies. (**A**) Ectopically expressed CUL4A didn’t bind with endogenous ZEB1. (**B**) Ectopically expressed CUL4B didn’t bind with endogenous ZEB1. (**C**) Ectopically expressed DDA1 didn’t bind with endogenous ZEB1. (**D**) Ectopically expressed DDB1 didn’t bind with endogenous ZEB1. (**E**) SMMC-7721 cells were transfected with the control or si-CUL1, si-CUL2, si-CUL3, si-CUL4B and si-CUL5 respectively. Cell lysates were analyzed by immunoblotting with the indicated antibodies. Expression of si-CUL1, si-CUL2, si-CUL3, si-CUL4B and si-CUL5 had no effect on ZEB1 degradation, in contrast, si-CUL4A promoted the ZEB1 protein expression.

Ubiquitin is conjugated to its substrates by Cullin RING ligases (CRLs), which are multi-subunit ubiquitin ligases. CRLs are modular complexes that contain a common catalytic core but assemble with a diverse set of receptors that recruit specific substrates to the CRL’s catalytic complex [6]. Within CRL4 (CUL4A and CUL4B), DDB1 and DDA1 are adaptors, while DCAF15 is a substrate-specific receptor [7]. We first tested whether CUL4A binds ZEB1 within cells. As shown in Figure 4A, ZEB1 was not immunoprecipitated by Flag-CUL4A, indicating no binding between ZEB1 and CUL4A (Figure 4A). Likewise, exogenous CUL4B did not bind ZEB1, nor did DDA1 or DDB1 (Figure 4B–4D). We only detected an interaction between endogenous ZEB1 and exogenous Flag-DCAF15 (Figure 2D). Moreover, when we knocked down endogenous CUL1, CUL2, CUL3, CUL4A, CUL4B or CUL5 using corresponding siRNAs in SMMC-7721 cells and then measured the levels of ZEB1 and Cullin family members, we found that when cells were transfected with siCUL4A, ZEB1 levels were significantly higher than control (Figure 4E). By contrast, transfection of siRNAs targeting other Cullin family proteins had no effect on ZEB1 levels. Thus, only CUL4A appears to affect the stability of ZEB1 protein.

### DCAF15 inhibits cell proliferation, migration and invasion by HCC cells

**Figure 5:**
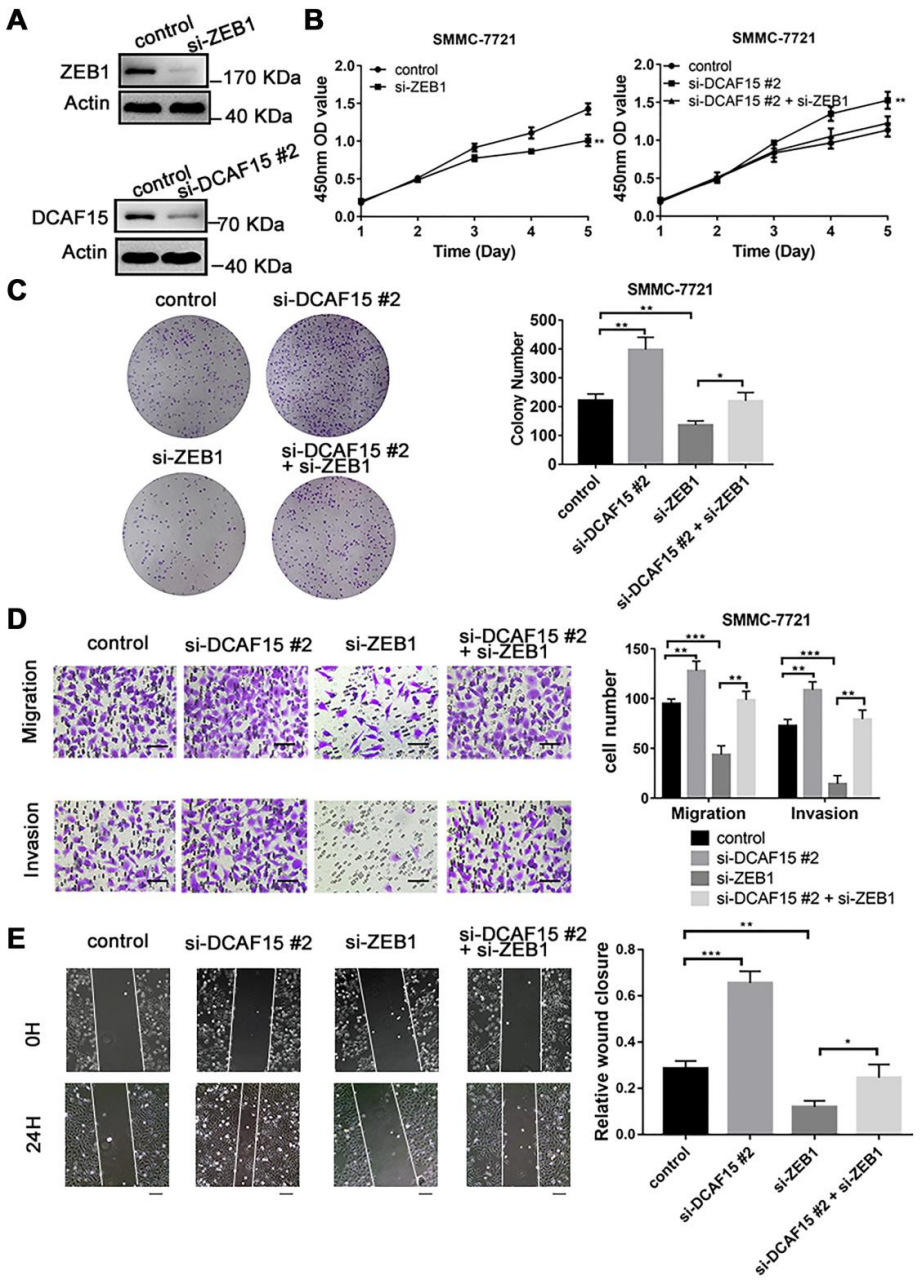
**DCAF15 inhibits proliferation, migration, and invasion of HCC cells.** (**A**) DCAF15 and ZEB1 was knocked down in SMMC-7721 cells by expressing siRNAs. Immunoblotting analyses were performed with the indicated antibodies. (**B**) CCK-8 assay was performed for cell proliferation of control, ZEB1-knockdown, DCAF15-knockdown and ZEB1-& DCAF15-knockdown SMMC-7721 cells. (**C**) The cells of (**B**) were plated and cultured on 6-well plates. Colony numbers were quantified. (**D**) The migration and invasion ability of the cells of (**B**) in were examined using the Transwell migration and invasion assay respectively. Cell numbers were quantified. Data are presented as mean ± SD (*n* = 3). ^**^P < 0.01, ^***^P < 0.001. Scale bars, 50 μm. (**E**) The cells of (**B**) were plated and cultured on 6-well plates. The cell layer was scratched with a 10 μl pipette tip. For each sample, at least three scratched fields were photographed immediately (0 h) or 24 h after scratching. Photographs of representative images were taken at ×100 magnification.

To explore the biological function of the DCAF15-ZEB1 axis in HCC, siRNAs targeting DCAF15 and ZEB1 were transiently transfected into SMMC-7721 cells (Figure 5A), after which CCK8 and colony formation assays were performed. The results showed that cell proliferation was inhibited by ZEB1 knockdown but promoted by DCAF15 knockdown. Moreover, the enhanced cell proliferation caused by DCAF15 knockdown could be reversed by ZEB1 co-knockdown (Figure 5B–5C). This suggests DCAF15 inhibits HCC cell proliferation.

We next performed transwell and wound healing assays to assess the impact of DCAF15 and ZEB1 on cell migration and invasion. We found that ZEB1 knockdown suppressed both migration and invasion by HCC cells, whereas DCAF15 knockdown had the opposite effect. Moreover, the enhanced migration and invasion caused by DCAF15 knockdown was reversed by ZEB1 co-knockdown (Figure 5D–5E), again demonstrating the functional interconnection of these two proteins.

### DCAF15 inhibits EMT in HCC cells

**Figure 6:**
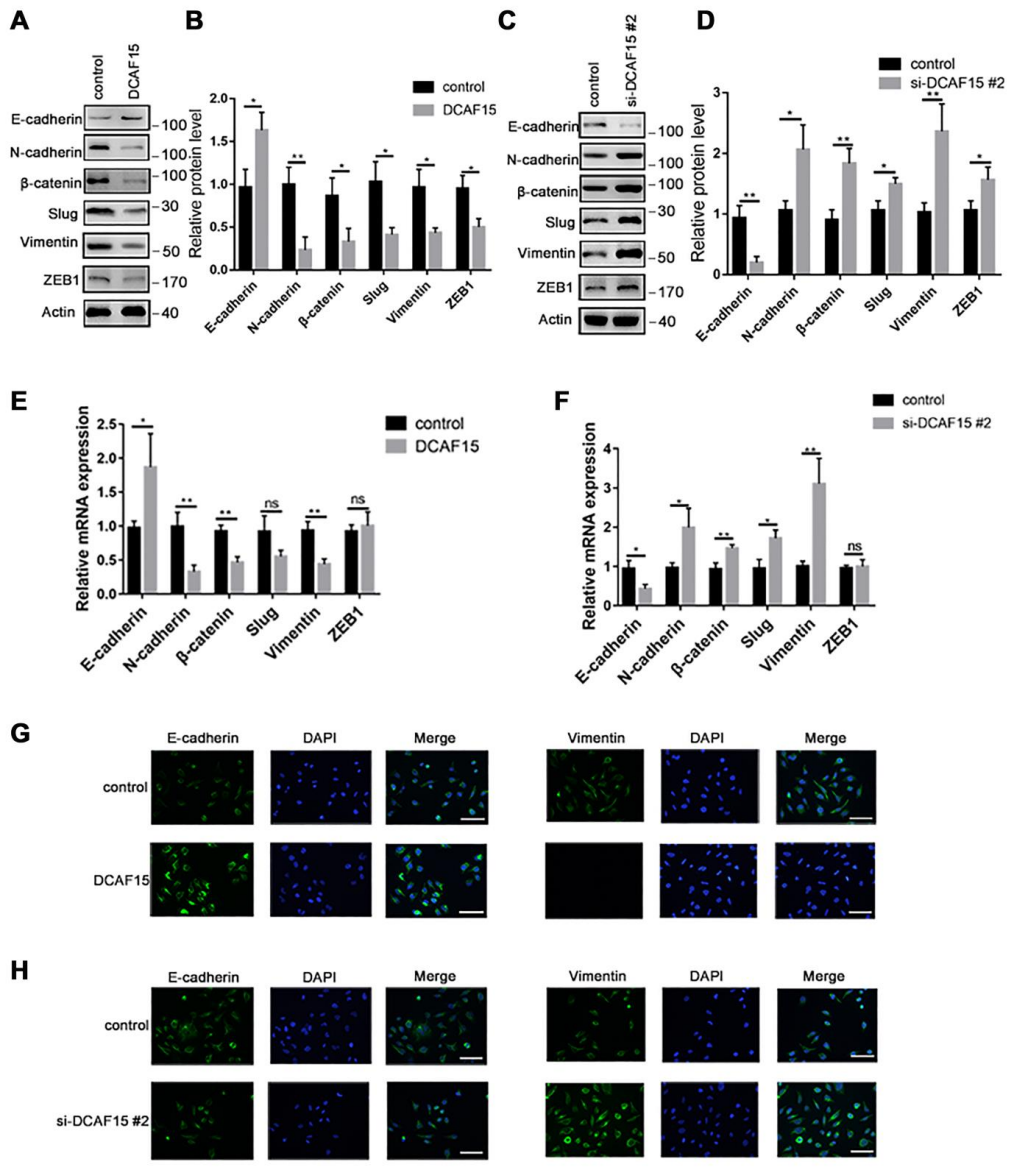
**DCAF15 inhibits epithelial-mesenchymal transition (EMT) of HCC cells**. (**A**–**B**) Western blot analysis showed the protein level of EMT markers after transfecting with control or DCAF15 overexpression plasmids in HCC cells. (**C**–**D**) Western blot analysis showed the protein level of EMT markers after transfecting with control or DCAF15 siRNA in HCC cells. (**E**) qRCR analysis showed relative mRNA level of EMT markers after transfecting with control or DCAF15 overexpression plasmids in HCC cells. (**F**) qRCR analysis showed relative mRNA level of EMT markers after transfecting with control or DCAF15 siRNA in HCC cells. (**G**) Protein level of E-cadherin and vimentin after transfecting with control or DCAF15 overexpression plasmids in HCC cells was visualized by fluorescence microscopy. Scale bars, 100 μm. (**H**) Protein level of E-cadherin and vimentin after transfecting with control or DCAF15 siRNA in HCC cells was visualized by fluorescence microscopy. Scale bars, 100 μm.

Numerous studies have reported that EMT is necessary for cancer metastasis [18, 19]. To become highly mobile, epithelial cells are known to lose their polarity, which helps them acquire migratory and invasive capabilities. Upon DCAF15 overexpression, E-cadherin was upregulated while several mesenchymal markers, including N-cadherin, β-catenin, slug and vimentin, were all downregulated (Figure 6A–6B, 6E and 6G). Conversely, E-cadherin was downregulated when DCAF15 expression was suppressed, and the mesenchymal signals were correspondingly upregulated (Figure 6C–6D, 6F and 6H).

### DCAF15 and ZEB1 are negatively correlated in HCC

**Figure 7:**
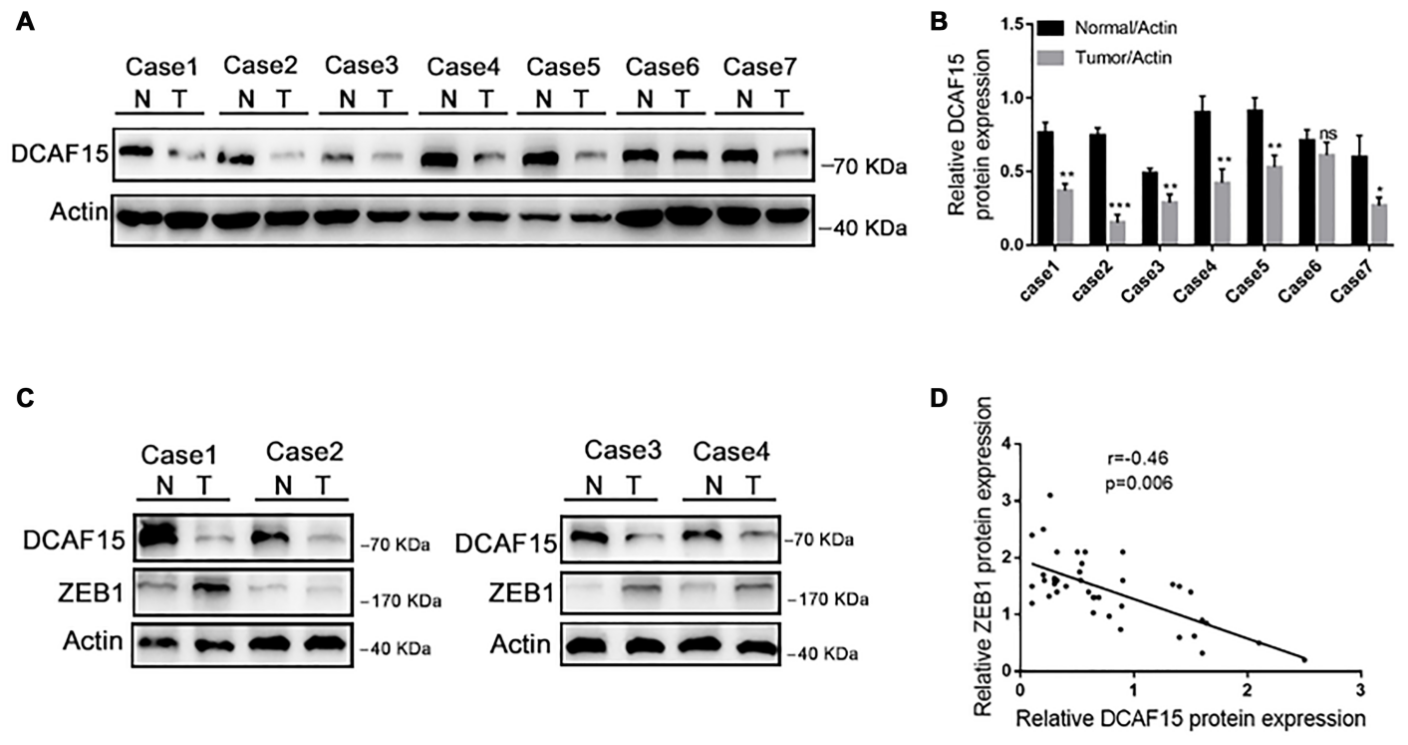
**DCAF15 and ZEB1 protein levels negatively correlate in HCC patient specimens.** (**A**–**B**) In random 7 HCC patient specimens. protein level of DCAF15 in tumor tissues is lower than in normal tissues. (**C**) Representative western blotting figures show protein expression of DCAF15 and ZEB1 in 40 HCC patient specimens. (**D**) Correlation analysis of the expression levels of DCAF15 and ZEB1 protein in 40 HCC patient specimens (*r* = −0.46, *p* = 0.006).

Finally, we assessed DCAF15 and ZEB1 expression in tissue specimens from 40 HCC patients. As shown in Figure 7A–7B, DCAF15 levels were lower in HCC than in paired normal liver tissues. In addition, DCAF15 and ZEB1 levels were negatively correlated in HCC tissues (*r* = −0.46, *p* = 0.006) (Figure 7C and 7D). We also used The Cancer Genome Atlas (TCGA) database to analyze the clinical prognostic significance of DCAF15 or ZEB1 mRNA expression. Kaplan-Meier analyses revealed that overall survival (OS) among HCC patients exhibiting low DCAF15 mRNA expression was poorer than among those exhibiting high DCAF15 mRNA expression, and that low DCAF15 or high ZEB1 mRNA expression was predictive of unfavorable OS in HCC patients (Supplementary Figure 1).

## DISCUSSION

**Figure 8:**
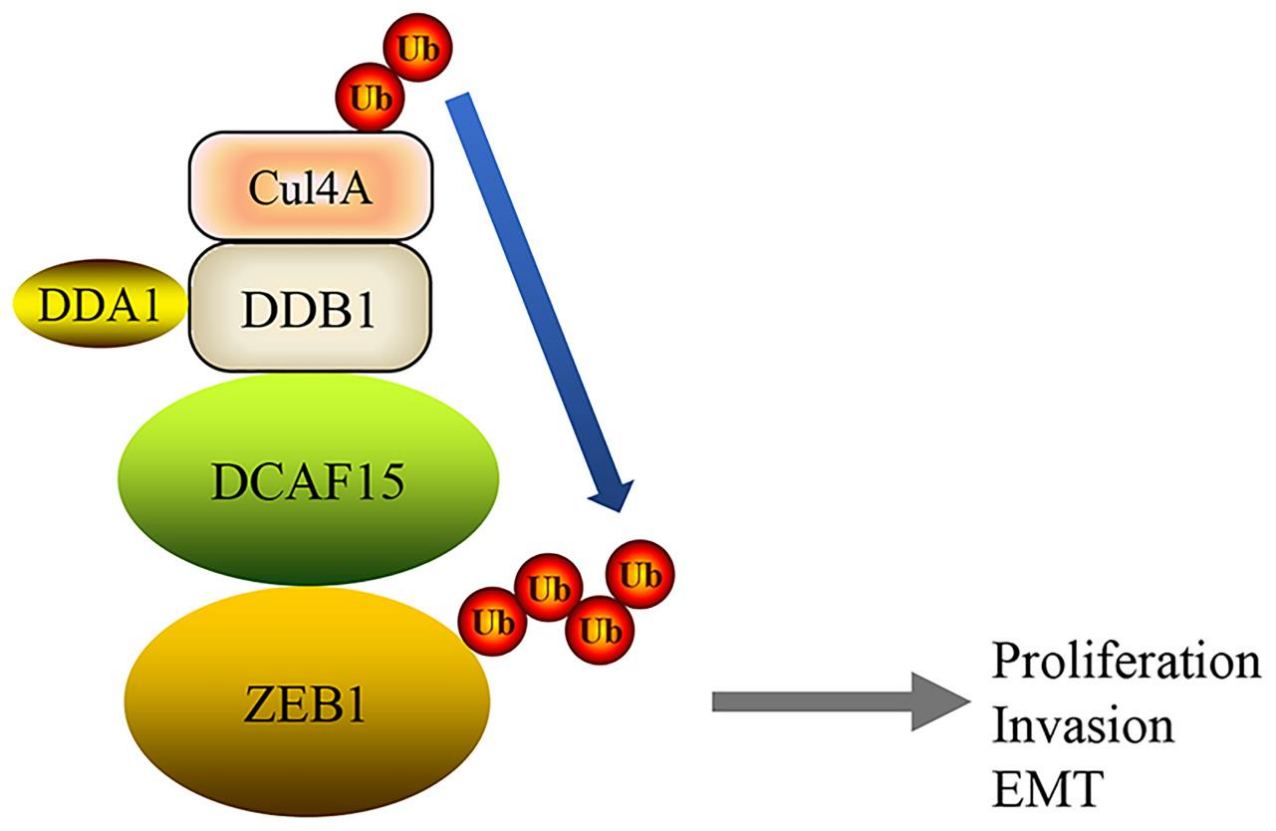
**A mechanism of DCAF15 function as a tumor suppressor through ubiquitination and degradation of ZEB1 leads to proliferation, invasion and EMT of HCC.**

In this study, we showed that the DCAF15-CUL4 E3 ligase complex functions as a tumor suppressor that promotes ZEB1 ubiquitination and degradation, which suppresses malignant phenotypes such as cell proliferation, migration and invasion as well as EMT in HCC cells (Figure 8).

ZEB1 is a key transcriptional regulator in EMT, which consists of two zinc finger clusters and a centrally-located homeodomain capable of DNA binding [20, 21]. Previous studies have shown that ZEB1 is highly expressed in HCC and that its overexpression promotes tumor cell migration, invasion and metastasis through EMT [22–26]. CUL4A, a member of the Cullin family and a component of the E3 ubiquitin ligase complex, is necessary for the ubiquitination and degradation of proteins [27]. Numerous studies have elaborated on Cullin family proteins and their associated CRLs E3 ligases [28–31]. Among them, CUL4 related E3 ubiquitin ligases comprise a Cullin 4 scaffold and the triple β-propeller DDB1 adaptor. However, few substrate receptors have been identified [32]. DCAF15 was first detected as a vital ubiquitin ligase contributing to the cytotoxicity of indisulam (a carbonic anhydrase inhibitor) -induced RBM39 degradation [7, 15]. A subsequent kinetic analysis found that aryl sulfonamide and RBM39 bind to DCAF15 in a synergistic manner [33], and it was later demonstrated that E7070, an aryl sulfonamide drug, mediates ubiquitination and proteasomal degradation of PRPF39 by recruiting PRPF39 to the CUL4-DCAF15 E3 ubiquitin ligase [34]. A variety of substrate receptors have been described as targets of DCAF15; however, the biological and functional significance of this ubiquitination event requires further elucidation. To our knowledge, this is the first report revealing the critical role of DCAF15-ZEB1 binding in HCC. Our findings suggest *DCAF15* serves as a tumor suppressor gene in HCC and is negatively related to ZEB1 protein. These results provide a mechanistic explanation for the clinical observation that HCC patients have high levels of ZEB1 in their tissues.

In summary, our study demonstrates that ZEB1 is recognized by DCAF15 as a substrate in human HCC cells and offers new insight into the specific functions of DCAF15 in HCC and a unique DCAF15-ZEB1 axis in the E3 ubiquitin ligase system.

## MATERIALS AND METHODS

### Cell culture

Human HCC cell lines (SMMC-7721 and Hep-3B) and HEK293T cells were obtained from the Type Culture Collection of the Chinese Academy of Sciences (Shanghai, China). They were cultured in high-glucose Dulbecco’s modified Eagle’s medium (DMEM) (Gibco) supplemented with 10% fetal bovine serum (FBS) (Gibco), 100 mg/ml penicillin and streptomycin (Gibco) at 37°C in a humidified atmosphere containing 5% CO_2_.

### Plasmids Constructs

The DCAF15 and ZEB1 cDNAs were purchased from Genechem and subcloned into pCMV-Flag or pCMV-Myc expression vectors, respectively. ZEB1 deletion mutants were generated by PCR, and subcloned into pCMV-Myc expression vectors. All the constructs were confirmed by DNA sequencing.

### RNA Interference

Human DCAF15 and Culling family siRNAs were purchased from GenePharma (Shanghai, China). siRNA transfection of cells was performed following the manufacturer’s instructions. The siRNA oligos sequences information is provided in Supplementary Table 1. The cells were transiently transfected with siRNAs using Lipofectamine 2000 (Invitrogen) according to the manufacturer’s instructions.

### Western blotting analysis

Cells were washed three times with 1× phosphate buffer saline (PBS) for 5 min, and then lysed with ice-cold radioimmunoprecipitation buffer containing a protease inhibitor cocktail (Sigma-Aldrich) for 30 min. And the lysate containing protein was centrifuged, then analysis with Western blot. Cell lysates or immune-precipitates were resolved by SDS-PAGE and proteins were transferred onto polyvinylidene difluoride membranes. The membranes were then maintained in 5% non-fat milk blocking for 2 h, and incubated with primary antibody for 12 h and followed by secondary antibody for 1h at room temperature. The proteins bands were visualized using ECL chemiluminescence system (Santa Cruz). The following antibodies were used: DCAF15 (SAB1103260, Sigma), ZEB1 (4C4, Novus), Myc (M4439; Sigma), Flag (F3165, Sigma), HA (M180-7; MLB), CUL1 (ab75817, Abcam), CUL2 (10981-2-AP, Proteintech), CUL3 (11107-1-AP, Proteintech), CUL4A (2715-S, EPITOMICS), CUL4B (12916-1-AP, Proteintech), CUL5 (ab34840, Abcam), and Actin (AC028; ABclonal). E-cadherin, N-cadherin, vimentin, β-catenin, and Slug antibodies were from CST (9782).

### Immuno-precipitation analysis

To immune-precipitate the ectopically expressed Flag-tagged proteins, transfected cells were lysed in NP40 buffer. The whole-cell lysates were immune-precipitated with anti-Flag antibody-conjugated M2 agarose beads (Sigma) at 4°C overnight. After three washes with Flag lysis buffer, followed by two washes with BC100 buffer, the bound proteins were eluted using Flag-Peptide (Sigma)/BC100 for 5 h at 4°C. To immune-precipitate the endogenous proteins, cells were lysed with RIPA buffer. The supernatant was precleared with protein A/G beads (Sigma) and incubated with indicated antibodies overnight. Thereafter, protein A/G beads were applied at 4°C overnight. After 2 h of incubation, pellets were washed five times with lysis buffer and resuspended in sample buffer. And analysis with Western blot.

### Immunofluorescence and confocal microscopy

Transfected cells were plated in glass-bottomed culture dishes (NEST) for 24 h. They were fixed with 4% paraformaldehyde, and then washed with PBS. After that, cells were permeabilized with 0.1% Triton X-100 for 15 min, and then washed with PBS. After treating the cells with blocking buffer (Beyotime) for 30 min, they were incubated with anti-Flag, anti-Myc, E-cadherin and vimentin, at 4°C overnight. Then, fluorescence labelled secondary antibodies were applied, and following DAPI was counterstained for 1 h at room temperature. An anti-fluorescence quencher was added dropwise and fixed with a coverslip. Images were taken using a confocal microscope (LSM710, Zeiss).

### Quantitative RT-PCR (qRT-PCR)

Total RNA was extracted from transfected cells using the TRIzol reagent (Invitrogen), and the concentration was measured by NanoDrop1000 Spectrophotometer (Agilent, USA). cDNA was reversed transcribed by the Superscript RT kit (TOYOBO), according to the manufacturer’s instructions. qRT-PCR amplification was performed using the SYBR Prime Script RT-qPCR kit (Takara, Japan). All quantization was normalized to the level of internal control GAPDH. The primer sequences for qRT-PCR were listed in Supplementary Table 1.

### CCK-8 assay

1,000 cells were plated in 96-well plates in 100 μl of media. 10 μl Cell Counting Kit (CCK-8) (Yeasen) solution was added into medium for 30 min before measuring absorbance at a wavelength of 450 nm by a microplate reader (Thermo Scientific) daily for continuous 5 days.

### Colony formation assay

Cells were seeded at 6-well plates with a density of 1 × 10^3^ cells per well and then cultured for in complete medium for 10 days. Then, the colonies were fixed using 4% methyl alcohol for 20 min and stained with 5% crystal violet solution. Clones were photographed and counted to evaluate cell proliferation ability.

### Migration and invasion assays

Migration and invasion chambers precoated with or without Matrigel (Corning) were prepared and placed into 24-well plates. Cells were digested into single cell suspension in 100 μl serum-free medium and 1 ×10^5^ cells were seeded into the upper chamber. And 700 μl DMEM medium with 10% FBS was added into the lower chamber. After 24 h of incubation, cells that invaded through the filters were stained with using 4% methyl alcohol for 20 min and stained with 5% crystal violet solution. The non-invading cells were wiped out with a cotton swab. The membrane was randomly photographed by a microscope.

### Wound healing assay

After transfected cells reaching 80% confluence, they were cultured with 1% fetal bovine serum for 12 h. The scratch was made by sterile pipette tips and mark the scratched point. The migration distance was photographed at 0 h and 24 h at the same location. The average migration distance was measured and analyzed.

### Statistical analysis

Student’s *t*-test was used for the two-group test, and one-way ANOVA was used for three or more groups test. Survival analysis was conducted using the Kaplan-Meier method. All data are shown as mean values ± SEM for experiments performed with at least three replicates. SPSS 22.0 and GraphPad Prism 6.0 software were used for analyses. ^*^ represents *p* < 0.05; ^**^ represents *p* < 0.01; ^***^ represents *p* < 0.001.

## Supplementary Materials

Supplementary Figure 1

Supplementary Table 1
