# Supplementary material for: Tumor suppressor DCAF15 inhibits epithelial-mesenchymal transition by targeting ZEB1 for proteasomal degradation in hepatocellular carcinoma
Source: Aging (Albany NY). 2021 Apr 4;13(7):10603–18. doi: 10.18632/aging.202823 (PMC8064142; doi:10.18632/aging.202823)
Supplement: Supplementary Figure 1 [file aging-13-202823-s001.pdf]

SUPPLEMENTARY FIGURE

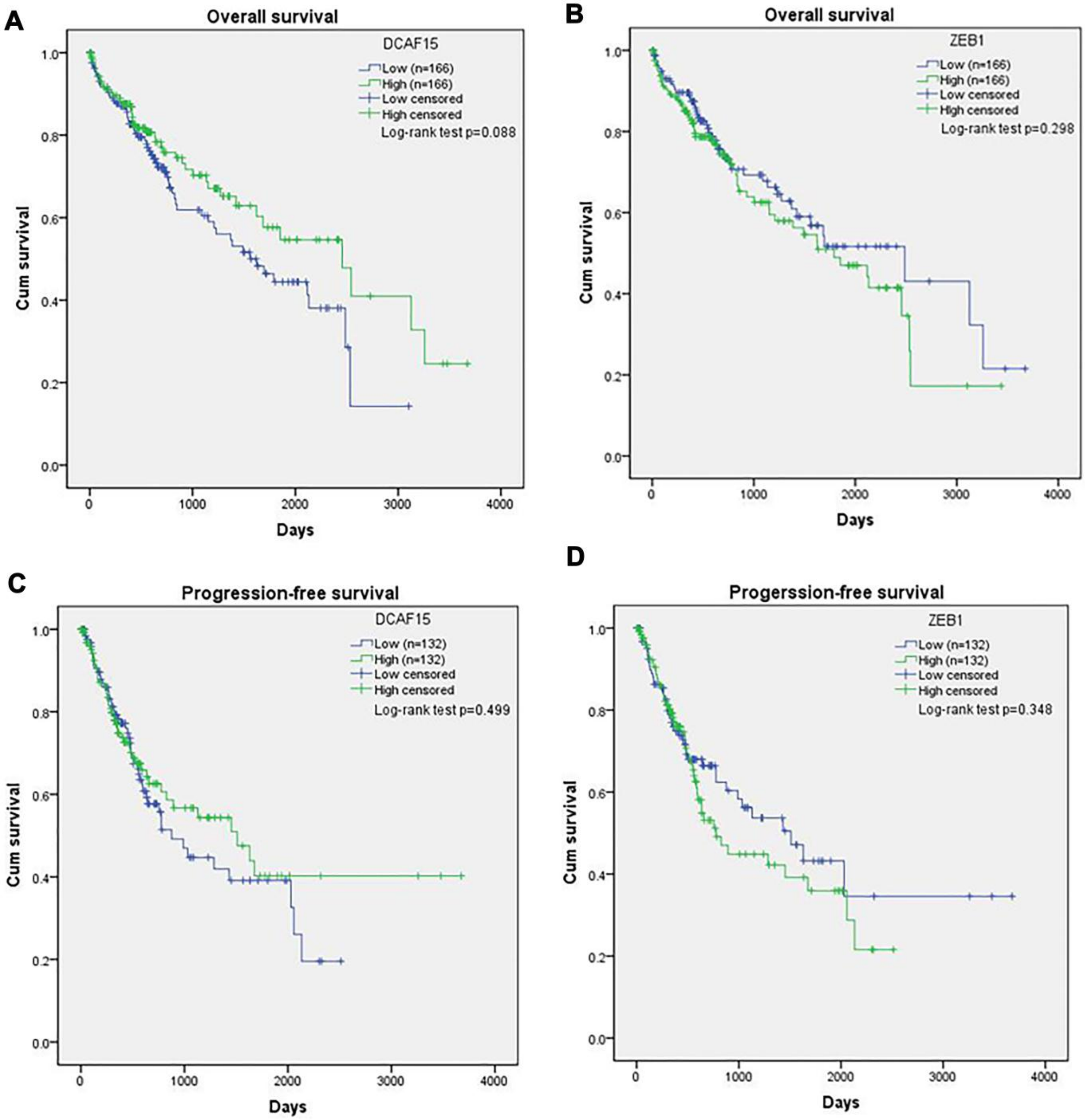

Supplementary Figure 1. DCAF15 was associated with better prognosis for TCGA HCC patients. Kaplan-Meier analysis of OS and DFS in HCC patients. OS (**A**–**B**) and DFS (**C**–**D**) of patients according to different DCAF15 and ZEB1 RNA expression.
