# Supplementary material for: Tumor suppressor DCAF15 inhibits epithelial-mesenchymal transition by targeting ZEB1 for proteasomal degradation in hepatocellular carcinoma
Source: Aging (Albany NY). 2021 Apr 4;13(7):10603–18. doi: 10.18632/aging.202823 (PMC8064142; doi:10.18632/aging.202823)
Supplement: Supplementary Table 1 [file aging-13-202823-s002.pdf]

SUPPLEMENTARY TABLE

Supplementary Table 1. Primers used for RT-qPCR in cultured cell lines, and sequences of siRNAs.

**Primers for RT-qPCR with cell lines samples**		
Gene name	F: 5′-3′	R: 5′-3′
DCAF15	CGAGCCTGGCTATGTCAACT	TCTTGTCGTCCTCCAACTCAT
ZEB1	AGCAGTGAAAGAGAAGGGAATGC	GGTCCTCTTCAGGTGCCTCAG
E-cadherin	GCTTCAGTTCCGAGGTCTAC	GCCAGTGCATCCTTCAAATC
N-cadherin	GTGGAGGCTTCTGGTGAA AT	GGCTCGCTGCTTTCATACT
β-catenin	CATCTACACAGTTTGATGCTGCT	GCAGTTTTGTCAGTTCAGGGA
vimentin	GAGGAGATGCTCCAGAGAGA	TCCTGC AAGGATTCCACTTT
Slug	TGATGCCCAGTCTAGGAAAT	AGTGAGGGCAAGAGAAAGG
GAPDH	GAAGGTGAAGGTCGGAGT	GAAGATGGTGATGGGATTTC
**Sequences of siRNAs**		
Gene name	Sequence	
si-DCAF15 #1	GGGUGUGCGUGUCCCUCAA	
si-DCAF15 #2	AGUUCAACGUUCACAGCAA	
si-ZEB1	CCUAGUCAGCCACCUUUAA	
siCUL1	CAACGAAGAGUUCAGGUUU	
siCUL2	GGAAGUGCAUGGUAAAUUU	
siCUL3	GAGAAGATGTACTAAATTC	
siCUL4A	GCACAGAUCCUUCCGUUUA	
siCUL4B	UAAAUAACCUCCUUGAUGA	
siCUL5	GACACGACGUCUUAUAUUA	
